# Using machine learning to assess the predictive potential of standardized nursing data for home healthcare case-mix classification

**DOI:** 10.1007/s10198-020-01213-9

**Published:** 2020-06-29

**Authors:** Maud H. de Korte, Gertjan S. Verhoeven, Arianne M. J. Elissen, Silke F. Metzelthin, Dirk Ruwaard, Misja C. Mikkers

**Affiliations:** 1 Dutch Healthcare Authority (NZa), Utrecht, The Netherlands; 2 Department of Economics, Tilburg University, Tilburg, The Netherlands; 3 Department of Health Services Research, Care and Public Health Research Institute (CAPHRI), Faculty of Health, Medicine and Life Sciences, Maastricht University, Maastricht, The Netherlands; 4 Tilburg Law and Economics Center (TILEC), Tilburg University, Tilburg, The Netherlands

**Keywords:** Case-mix, Home care, Electronic health records, Machine learning, Predictive modelling, I13 health insurance, Public and private, I11 analysis of health care markets, I18 government policy, Regulation, Public health, C53 forecasting and prediction methods, Simulation methods

## Abstract

**Background:**

The Netherlands is currently investigating the feasibility of moving from fee-for-service to prospective payments for home healthcare, which would require a suitable case-mix system. In 2017, health insurers mandated a preliminary case-mix system as a first step towards generating information on client differences in relation to care use. Home healthcare providers have also increasingly adopted standardized nursing terminology (SNT) as part of their electronic health records (EHRs), providing novel data for predictive modelling.

**Objective:**

To explore the predictive potential of SNT data for improvement of the existing preliminary Dutch case-mix classification for home healthcare utilization.

**Methods:**

We extracted client-level data from the EHRs of a large home healthcare provider, including data from the existing Dutch case-mix system, SNT data (specifically, NANDA-I) and the hours of home healthcare provided. We evaluated the predictive accuracy of the case-mix system and the SNT data separately, and combined, using the machine learning algorithm Random Forest.

**Results:**

The case-mix system had a predictive performance of 22.4% cross-validated *R*-squared and 6.2% cross-validated Cumming’s Prediction Measure (CPM). Adding SNT data led to a substantial relative improvement in predicting home healthcare hours, yielding 32.1% *R*-squared and 15.4% CPM.

**Discussion:**

The existing preliminary Dutch case-mix system distinguishes client needs to some degree, but not sufficiently. The results indicate that routinely collected SNT data contain sufficient additional predictive value to warrant further research for use in case-mix system design.

**Electronic supplementary material:**

The online version of this article (10.1007/s10198-020-01213-9) contains supplementary material, which is available to authorized users.

## Introduction

As part of the far-reaching reform of long-term care (LTC) in 2015, home healthcare financing in the Netherlands shifted from a public insurance scheme to the pre-existing mandatory health insurance scheme, which is administered by private health insurers [1, 2]. The rationale behind this reform was to improve the coordination of care and enable more efficient care by making competing and risk-bearing health insurers responsible for contracting home healthcare [1]. When the LTC reform was implemented, no case-mix system was in place for home healthcare. Claims were settled on a fee-for-service (FFS) basis and only informative of the number of care hours provided, giving insurers little insight into differences between clients in relation to their use of care. As a first step towards generating this information, health insurers introduced a preliminary system of seven case-mix groups [3]. As of 2017, this constitutes the case-mix system for insurer-procured home healthcare in the Netherlands.

The Netherlands is currently investigating the feasibility of transitioning from FFS to a prospective payment system, such as the Home Health Prospective Payment System used by Medicare in the United States (US) [4, 5], in order to improve incentives. Since setting payments prospectively could incentivize providers to engage in risk selection, adjusting payment rates for client heterogeneity in resource use is crucial in order to create and maintain a level playing field for home healthcare providers [6, 7]. In home healthcare, the use of resources is typically determined by a wide number of factors, including physical and psychosocial functioning as well as the presence of a social network [8–10]. These factors are only partially included in the existing Dutch case-mix system.

Existing home healthcare case-mix systems tend to use a dedicated assessment instrument or survey to collect information on client characteristics for classification purposes [11–13]. However in recent years, home healthcare providers in many countries, including the Netherlands and the US, have increasingly adopted standardized nursing terminology (SNT) as part of their electronic health records (EHRs). SNTs allow nurses to document the process of providing home healthcare services in a uniform manner, by describing clients’ problems, interventions and outcomes [14]. Currently, 7 SNTs are recognized by the American Nurses Association (ANA) [15]. As indicated by a review of the scientific literature, NANDA-I and Omaha System are the most widely studied SNTs [15]. Deriving a case-mix system based on an SNT obtained from providers’ EHRs would have several advantages, such as minimizing the administrative burden and promoting the meaningful use of EHR data [16–18].

Developing a case-mix system involves many design choices. An ideal case-mix system would predict resource use perfectly, classify clients with similar diagnoses and needs into similar categories and be immune to strategic behavior such as upcoding [12, 19]. Of these requirements, predictive accuracy is the most essential and, arguably, should be evaluated first, before considering the other requirements. The objective of this study was, therefore, to explore the predictive potential of SNT data for improvement of the existing preliminary Dutch case-mix classification for home healthcare utilization. We used the Random Forest machine learning algorithm to estimate the upper bound of the predictive value of the SNT as a whole. To our knowledge, this study is the first to assess the potential gains of using standardized nursing data with the aim of predicting the use of home healthcare.

## Methods

### Setting

Home healthcare services in the Netherlands include both district nursing care and personal care services. District nurses assess the type and level of clients’ needs and use an SNT to support this. The most widely used SNTs within the Netherlands are NANDA-I and Omaha System [20].

### Data source

The data used for this study were obtained from the EHRs of MeanderGroep, a large home healthcare provider in the Netherlands. Two-thirds of the total expenses for home healthcare are accounted for by 52 providers, including the provider involved in this study [21]. The remaining share comes from around 2000–3000 smaller home healthcare providers or self-employed nurses. In 2016, the provider agreed to implement an experimental payment system involving the two largest health insurers in the region (with a market share of around 80–90%, based on publicly available market share data from 2014 [22]). Under the experimental payment system, the insurers pay the provider fixed monthly fees per client. Clients who are insured elsewhere are subject to the regular FFS system. The provider used the SNT NANDA-I for the needs assessments carried out by district nurses. NANDA-I structures over 200 nursing diagnoses in 13 different domains of nursing practice [23]. Diagnoses include existing health problems, risk states and readiness for health promotion. District nurses use symptoms and etiological factors or risk factors to determine which diagnoses apply.

### Study sample

**Fig. 1:**
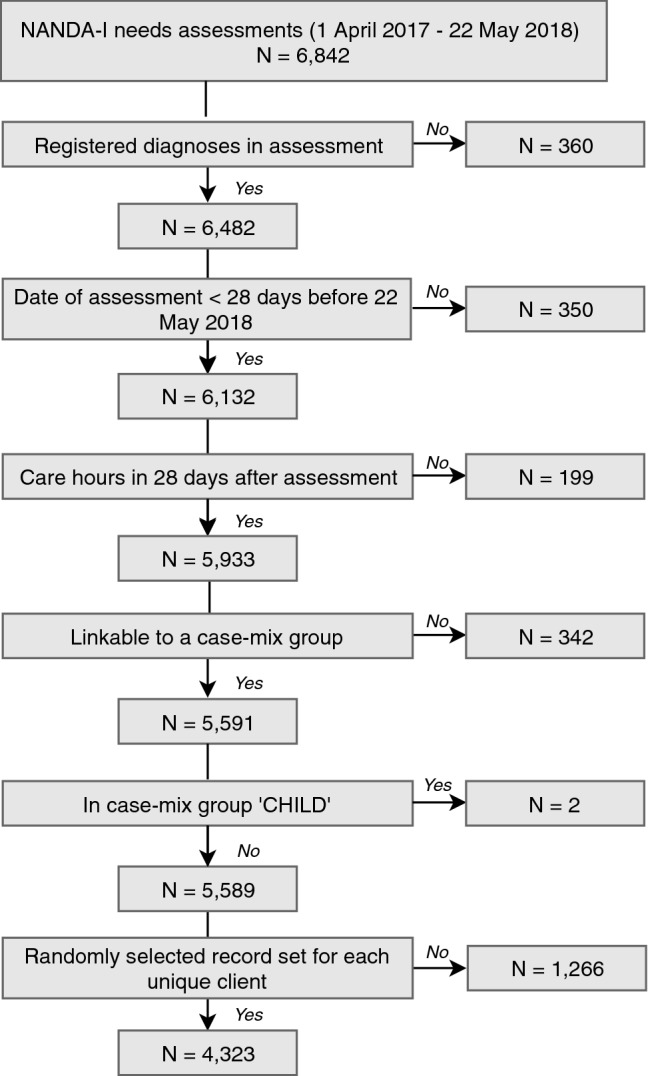
Flowchart of the study sample selection process

The starting point for the dataset was all clients with NANDA-I characteristics that received home healthcare between April 1st 2017 up to May 22nd 2018 (cut-off point of the data obtained). These clients received 90% of the registered home healthcare activities by the provider during that period. In addition, we required that the NANDA-I assessment was created anew during this time period. This resulted in 6,842 sets of client records, each corresponding to a needs assessment between April 2017 and May 22nd 2018. Figure 1 depicts the study sample selection process from this point onwards (see Supplementary Material 1 for additional information on the selection process).

Client record sets without registered NANDA-I diagnoses (*N* = 360), with incomplete 28-day post-assessment period as a result of the cut-off point of the data (*N* = 350) and without any care hours in the 28-day post-assessment period (*N* = 199) were excluded from the analyses Supplementary Material 2 provides the characteristics of the clients excluded from the study sample.

Each client record set included the NANDA-I characteristics that were registered during the needs assessment and was supplemented with the demographic factors of age, sex and marital status and the registered case-mix group. During the needs assessment, the district nurse determines which case-mix group is the most applicable to each client (see “Supplementary Material 3” for the decision tree that supports this selection process). The district nurse can choose between the following seven case-mix groups:

1. Care after hospital discharge or specialized nursing care < 3 months (ST-H)
2. Care for frail elderly and chronically ill < 3 months (ST-F)
3. Care for frail elderly and chronically ill > 3 months with a focus on somatic complaints (LT-SOM)
4. Care for frail elderly and chronically ill > 3 months with a focus on psychogeriatric complaints (LT-PG)
5. Preventive care for frail elderly without current care need (PREV)
6. Care for terminally-ill clients (PALL)
7. Care for children (CHILD)

Client record sets without a registered case-mix group were excluded from the analyses (*N* = 342). The case-mix group CHILD was only applicable to two client record sets, which were, therefore, also excluded.

Several clients were reassessed during the period of our study, due to changes in their health status for instance and, therefore, accounted for multiple assessments in our dataset. Including multiple assessments of one client would have led to multiple client records in the dataset that are likely very similar with respect to their SNT diagnoses. In the cross-validation (cv) procedure, these similar records could end up in both the train and test set and result in too optimistic out-of-sample accuracy. Therefore, only one record set per client was selected at random and retained in our dataset.^1^ This excluded a further 1266 client record sets. Our final dataset contained 4323 sets of client records, each concerning a unique client.

### Variables

Our outcome measure was the average weekly home healthcare hours per client. We defined a period of 28 days counting from the date of the NANDA-I needs assessment. All care hours provided within the 28-day period were allocated to a week number ranging from 1 to 4 based on the day on which they occurred. We then divided the total number of care hours by the number of individual weeks in which the client received care.

The variables included in each client record set were as follows:

1. Demographic factors age, sex and marital status (abbr. DEMO)
2. Case-mix groups (abbr. CM)
3. NANDA-I nursing diagnoses, symptoms and etiological/risk factors (abbr. NANDA-I)

The NANDA-I characteristics accounted for 3326 different variables in our dataset. To minimize the computational power required, we reduced the number of NANDA-I characteristics by selecting only those registered for at least 5 percent of all clients within a case-mix group. As a result, 388 NANDA-I characteristics remained in our dataset.

### Statistical analyses

We examined our data for possible erroneous values. Within each case-mix group, we checked the raw data pattern in the 28-day post assessment period for the top 1% of the records with highest average number of home healthcare hours. No questionable records were found that required exclusion from the analyses. As we analyzed the data cross-sectionally, we visually checked the stability of our variables during the period of our study and observed a fairly stable pattern (as shown in Supplementary Material 4–6).

We determined the predictive accuracy of the different sets of variables on home healthcare use using models built with the Random Forest algorithm [24]. Random Forest models are to a large extent immune to the presence of irrelevant variables in the dataset. However, if more irrelevant variables are added to a Random Forest model, at some point its performance will be adversely affected [25, 26]. We, therefore, performed recursive feature elimination (RFE) for all models in order to select relevant variables [27] (see Supplementary Material 7 for details on the methodology used including parameter tuning). Since our dataset consisted mainly of binary variables, the primary added value of the Random Forest over regression (ordinary least squares; OLS) was its ability to automatically capture interactions between variables. We verified that Random Forest performs better than OLS (results are shown in Supplementary Material 8).

Analyses were performed using R, using the Random Forest implementation in the package ranger (version 0.10.1) [28] and the package caret (version 6.0–80) for the model training process [29].

We performed analyses on the whole study sample and on subsets of our sample stratified using the case-mix groups. An intercept-only model (INT) that predicts the overall mean home healthcare hours was used as a baseline to evaluate all the other models. We then estimated a model containing the available demographic factors. In all subsequent models, the demographic factors were included as control variables. We estimated three additional models for the total study sample: (1) DEMO + CM; (2) DEMO + NANDA-I; and (3) DEMO + CM + NANDA-I. For the sample subsets per case-mix group, we estimated 1 additional model: (1) DEMO + NANDA-I. For each model, tenfold cv was applied in order to validate stability and to prevent overfitting. For the total study sample models, single tenfold cv was used. For the separate case-mix group models, tenfold cv was repeated 30 times and averaged for more precise error estimates and to quantify their standard deviation.

We compared the predictive performance of the models using the Mean Absolute Prediction Error (MAPE), the Cumming’s Prediction Measure (CPM), the Root Mean Squared Error (RMSE) and the *R*-squared (see Supplementary Material 9 for their definitions). CPM and *R*-squared are standardized measures for which higher values represent smaller error. For MAPE and RMSE, lower values indicate a better fit. RMSE and *R*-squared give more weight to larger errors, which makes them more sensitive to extreme values in the distribution. MAPE and CPM weight all errors equally and are, therefore, less affected by large errors.

## Results

Table 1 shows the specification of our study sample. Clients had on average 3 NANDA-I nursing diagnoses, 12 symptoms and 4 etiological factors or risk factors. The majority of clients were classified in the LT-SOM case-mix group, and the case-mix groups indicating short-term care need (ST-H and ST-F) were the next largest. Clients received on average 3 care hours per week. Case-mix group PALL included clients with the overall highest intensity of home healthcare hours. PALL also showed the largest variation in care hours.

**Table 1:** Study sample specifications

Parameter	Class	mean ± sd or *N*(%)	5th pctl	95th pctl
Demographic factors				
Age		75.8 ± 13.9	50	92
Gender	Male	1598 (37.0)		
	Female	2725 (63.0)		
Marital status	Unknown	2844 (65.8)		
	Unmarried	200 (4.6)		
	Married	570 (13.2)		
	Divorced	24 (0.6)		
	Widow(er)	677 (15.7)		
	Registered partnership	8 (0.2)		
Case-mix group	PREV	30 (0.7)		
	ST-H	694 (16.1)		
	ST-F	754 (17.4)		
	LT-SOM	2,359 (54.6)		
	LT-PG	389 (9.0)		
	PALL	97 (2.2)		
NANDA-I characteristics	Diagnoses	3 ± 3	1	9
	Symptoms	12 ± 13	1	38
	Etiologic/risk factors	4 ± 4	1	11
Weekly home care hours	Average	3.0 ± 5.4	0.4	7.9
	PREV	1.2 ± 1.3	0.2	4.5
	ST-H	1.8 ± 2.0	0.4	4.4
	ST-F	1.9 ± 2.3	0.4	4.8
	LT-SOM	3.0 ± 3.9	0.4	8.0
	LT-PG	3.1 ± 3.6	0.5	7.5
	PALL	20.7 ± 22.5	0.7	65.1

*sd* standard deviation, *ptcl* percentile

**Fig. 2:**
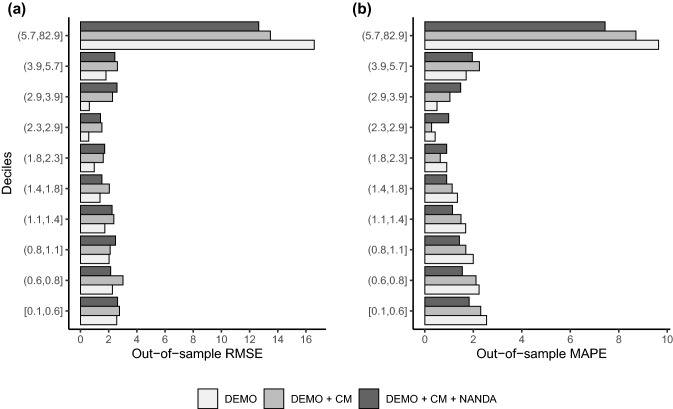
Fit results per decile of total study sample. **a** RMSE and **b** MAPE within each decile (sorted by observed home healthcare hours) for the models DEMO, DEMO + CM, DEMO + CM + NANDA-I)

The fit results for the total study sample models are shown in Table 2. Adding the case-mix groups to the demographic factors model improved performance on all measures. This was caused by the palliative case-mix group, as this was the only variable retained by the RFE procedure. The *R*-squared showed the greatest improvement: the DEMO model yielded an *R*-squared of − 0.1% and when including the case-mix groups the *R*-squared improved to 22.4%. The DEMO + CM model mainly improved the predictions for clients with highest number of care hours (shown in Fig. 2a), which positively influenced the *R*-squared. Adding the NANDA-I characteristics to the demographic factors model also improved the performance on all measures. The CPM was 0.3% in the DEMO model and 11.5% when the NANDA-I characteristics were included. The *R*-squared was − 0.1% in the DEMO model and improved to 15.2% in the DEMO + NANDA-I model. The DEMO + NANDA-I model mainly led to improvements in the predictions for clients in the lower deciles (shown in Fig. 2b), positively affecting the CPM. The model including all the selected variables (DEMO + CM + NANDA-I) performed the best, with an R-squared of 32.1% and CPM of 15.4% and an absolute prediction error of 2.0 h. This model made predictions up to 40 weekly home healthcare hours, whereas the dataset contained clients with higher intensity of care use. For example, this applied to 24% of the clients within the palliative case-mix group. Figure 2a and b also indicates that the largest prediction errors were in the upper decile, although the largest improvements achieved by adding the case-mix groups and NANDA-I characteristics were also here. These improvements came partly at the expense of the prediction errors in the lower deciles.

**Table 2:** Summary of fit results for total study sample

Model	MAPE (h)	CPM (%)	RMSE (h)	*R*-squared (%)
INT	2.3	0.0	5.4	0.0
DEMO	2.3	0.3	5.4	− 0.1
DEMO + CM	2.2	6.2	4.7	22.4
DEMO + NANDA-I	2.0	11.5	4.9	15.2
DEMO + CM + NANDA-I	2.0	15.4	4.4	32.1

*MAPE* mean absolute prediction error, *CPM* Cumming’s prediction measure, *RMSE* root mean squared error, *h*, hours

The predictive value of the NANDA-I characteristics was also assessed within each case-mix group. Table 3 shows the prediction accuracy for these models. PREV and PALL are not displayed, as we ran a risk of obtaining spurious correlations for these case-mix groups due to the large number of variables and small number of observations (see Supplementary Material 10 for the results for PREV and PALL). The largest MAPE was observed in case-mix group LT-SOM, for which adding NANDA-I led to the relatively largest reduction of the MAPE compared to the DEMO model. Also case-mix group ST-F benefited largely from the addition of NANDA-I. The R-squared increased to 13.8% hours in the NANDA-I model. The largest CPM and R-squared were both observed within case-mix group LT-SOM.

**Table 3:** Summary of fit results for study sample stratified by case-mix group

Case-mix group	Model	N clients	MAPE (h)	CPM (%)	± sd^*^ (%)	RMSE (h)	*R*-squared (%)	± sd^*^ (%)
ST-H	INT	694	1.0	0	0	2.0	0	0
	DEMO		1.0	− 0.6	0.3	1.7	− 1.7	0.4
	DEMO + NANDA-I		1.0	− 0.5	0.4	1.7	− 1.9	1.1
ST-F	INT	754	1.2	0	0	2.3	0	0
	DEMO		1.2	− 1.3	0.3	2.0	− 1.7	0.6
	DEMO + NANDA-I		1.1	9.4	0.6	1.9	13.8	2.1
LT-SOM	INT	2359	2.1	0	0	3.9	0	0
	DEMO		2.1	0.5	0.1	3.7	0.1	0.1
	DEMO + NANDA-I		1.8	15.4	0.3	3.4	17.5	1.1
LT-PG	INT	389	2.0	0	0	3.6	0	0
	DEMO		2.0	− 0.8	0.6	3.0	− 3.6	1.6
	DEMO + NANDA-I		1.8	6.6	0.7	2.9	8.4	1.4

*MAPE* mean absolute prediction error, *CPM* Cumming’s prediction measure, *RMSE* root mean squared error, *h* hours, *sd* standard deviation

*Standard deviation is generated using 30 repeated tenfold cross-validation

## Discussion

In this study, we have assessed the existing Dutch case-mix system for home healthcare and found a predictive performance of 6.2% cross-validated CPM and 22.4% cross-validated *R*-squared. The predictive performance of the system is determined by its ability to distinguish between palliative and non-palliative care. Adding SNT data (NANDA-I) obtained from EHRs to the case-mix groups led to 15.4% CPM and 32.1% *R*-squared, thus leading to a substantial relative improvement in predicting home healthcare hours. This was confirmed by the per case-mix group results which showed improvements in the predictions for all case-mix groups, except for case-mix group ST-H. While the degree of improvement differed between case-mix groups, the case-mix groups somatic long-term care need (LT-SOM) and short-term care need for frail elderly (ST-F) showed the greatest improvement in predictive accuracy when client characteristics from NANDA-I were added.

In order to assess the predictive performance of our models in a normative way, we compared the results with existing literature on case-mix systems that aim to predict home healthcare resource utilization. The *R*-squared values reported in related studies range from 16 to 54%. New Zealand’s Home & Community Support Services (HCSS) case-mix system explains 16 to 24% (*R*-squared) of variation in formal home healthcare hours [13]. These results were obtained using in-sample estimates, a method which is likely to overestimate the predictive performance due to overfitting, since the model is evaluated on the sample also used for fitting. For the Resource Utilization Groups version III Home Care (RUG-III/HC) model used in Canada, *R*-squared for formal home healthcare costs was 20.5% [12]. For Medicare’s Home Health Resource Groups (HHRGs), *R*-squared values ranging from 32% up to 54% have been reported [11, 30, 31]. The HHRGs are not fully prospective; however, because resource utilization (i.e. the number of therapy visits) is used to determine payment rates [30]. The inclusion of a parameter relating to resource utilization is known to enhance the performance of the model but could also incentivize the provision of excess care [30, 32]. Overall, this suggests that the performance of our predictive models is similar to models in related studies. However, these comparisons are weakened by dissimilarities relating to (1) the scope of home healthcare services, (2) predicting costs versus care hours, (3) the length of the care episode predicted and (4) the sample size.

In the Netherlands, SNTs have increasingly been adopted by home healthcare providers, which has accelerated the availability of SNT data within EHRs. However, a number of conditions must be fulfilled if this data is to be used for case-mix classification, the first being the standardization of an SNT. As in the US [15], no national SNT standard has yet emerged in the Netherlands, with 2 SNTs (NANDA-I and Omaha System) having large market shares. In the US, current policy objectives focus on interoperability between EHRs in hospitals and post-acute care, such as home healthcare. This also requires the standardization of nursing terminology, which could increase the feasibility of using SNT information for reimbursement purposes in the future. Second, the challenges and opportunities of linking financial incentives to SNT registration will require careful consideration. Since clients’ needs assessments lead directly to the provision of home healthcare, characteristics within SNT data are likely to be important cost drivers and, therefore, useful for a case-mix system. There is, however, also a risk of financial compensations influencing the SNT registration [33]. Although such perverse incentives apply to any instrument used for case-mix classification, in this case gaming would directly affect the primary source of information on the care process. This could, conversely, also raise the threshold to alter the registration of SNT in an unfair matter.

One strength of our study is that the method applied allows for the rapid screening of large groups of variables for their predictive value, without laborious manual model selection procedures. Also, the *R*-squared measure currently dominates the literature on predictive modelling. However, a small number of large prediction errors can greatly affect the *R*-squared value. Given the typically skewed distribution of healthcare resource utilization, using alternative performance measures in addition to the *R*-squared is informative. The CPM uses the absolute prediction errors and thus is less sensitive to large errors (i.e. outliers) [34]. Another strength is the re-use of SNT data as input for a case-mix system, which does not impose any additional administrative burden on home healthcare providers, and anticipates the increasing availability of EHRs at care providers.

This study also has several limitations. First, the home healthcare provider involved in our study mainly provided care under a prospective payment system. Although this, in the absence of an adequate case-mix correction, could lead to client selection, we have no indication that this was occurring in this case. Second, the data used in this study reflects all nursing diagnoses, symptoms and etiological factors that district nurses deemed necessary to register for their clients. Although all nurses have been trained to work with NANDA-I, variations in how NANDA-I is applied by different district nurses may exist. Last, our outcome measure of average weekly home healthcare hours does not distinguish between district nursing care and personal care services. Since this ratio might be indicative of differences in clients’ home healthcare needs, differentiated predictions could be required.

Due to its FFS elements, the existing payment system for the Dutch home healthcare market impedes the coordination of care and prevention while rewarding high volumes of care. It is for this reason that reform of the payment system to include prospective elements is high on the policy agenda. A robust case-mix system that adjusts for client differences will be vital when payments for home healthcare providers are set prospectively [6, 35]. We conclude that the existing Dutch case-mix system differentiates between client needs to a certain extent, but not sufficiently. To increase its role in home healthcare contracting, the predictive accuracy of the case-mix system should be further enhanced. Data from SNTs does indeed have sufficient predictive value to further explore its use within case-mix system design for home healthcare.

A central question in further research should be if the predictive value added by the SNT data justifies the efforts of collecting and incorporating this data in a case-mix system used for a nationwide payment system. This requires a trade-off between the complexity and associated (in)transparency of the case-mix system on the one hand and predictive accuracy of the system on the other hand. Also, a SNT based case-mix system should perform well in a range of diverse home healthcare settings when it is to be used as a national payment system. The results of this study might nog fully generalize to other home healthcare providers. The obtained results must, therefore, be assessed in other home healthcare settings to evaluate their generalizability. Clustering methods need to be applied that incorporate these relevant variables selected from the SNT data to create new case-mix groups. In order to develop a scientifically grounded case-mix system, future research should also include an assessment of whether the variables used for clustering are susceptible to gaming and an assessment of the clinical value of the resulting case-mix classification.

## Electronic supplementary material

Below is the link to the electronic supplementary material.

Supplementary file1 (DOCX 273 kb)

## Footnotes

1 We checked our assumption by performing the analyses for all client record sets, and indeed found higher predictive performance for all models compared to models only containing one record set per client.
