# Supplementary material for: Using machine learning to assess the predictive potential of standardized nursing data for home healthcare case-mix classification
Source: Eur J Health Econ. 2020 Jun 29;21(8):1121–9. doi: 10.1007/s10198-020-01213-9 (PMC7561562; doi:10.1007/s10198-020-01213-9)
Supplement: Supplementary file 1 — Supplementary file1 (DOCX 273 kb) [file 10198_2020_1213_MOESM1_ESM.docx]

**Electronic Supplementary Material**

**Article title**

“Using machine learning to assess the predictive potential of standardized nursing data for home healthcare case-mix classification”

**Journal name**

European Journal of Health Economics

**Authors**

Maud Hortense de Korte; Gertjan Sebastiaan Verhoeven; Arianne Mathilda Josephus Elissen; Silke Friederike Metzelthin; Dirk Ruwaard; Misja Chiljon Mikkers

**Corresponding author information**

Maud Hortense de Korte

Dutch Healthcare Authority (NZa); Department of Economics, Tilburg University

m.h.dekorte@uvt.nl

**Supplementary Material 1** Study sample selection process

**Table S1** Step-by-step study sample selection process

| Clients that received home healthcare between 1 April 2017 and 22 May 2018  N = 10,074 | | | |
| --- | --- | --- | --- |
| Inclusion criteria | | N included | N excluded |
|  | Clients that received NANDA assessment at least once | 6,919 | 3,155 |
|  | Clients with one ore more NANDA assessment(s) between 1 April 2017 and 22 May 2018 | 5,160 | 1,759 |
| *Included unique clients* | | *5,160* |  |
| *Included unique client record sets – corresponding to Figure 1 in the article* | | *6,842* |  |
| Exclusion criteria | |  |  |
|  | No registered diagnoses in NANDA-I assessment |  | 360 |
|  | Date of NANDA-I assessment < 28 days before cut-off point data |  | 350 |
|  | No care hours in 28 days after NANDA-I assessment |  | 199 |
|  | No registered case-mix group |  | 342 |
|  | In case-mix group ‘CHILD’ |  | 2 |
|  | Total |  | 1,253 |
| *Included unique client record sets* | | *5,589* |  |
| *Included unique clients / randomly selected client record sets for each unique client* | | *4,323* |  |

**Table S2** Total excluded client records per exclusion criteria

| Shown in | N client records | Exclusion criteria | | | | |
| --- | --- | --- | --- | --- | --- | --- |
|  |  | No registered NANDA diagnoses in NANDA-I assessment | Date of NANDA-I assessment < 28 days before cut-off point data | No care hours in 28 days after NANDA-I assessment | No registered case-mix group | In case-mix group ‘CHILD’ |
| *Table S5* | 342 | 0 | 0 | 0 | 1 | 0 |
| *Table S6* | 282 | 1 | 0 | 0 | 0 | 0 |
| *-* | 274 | 0 | 1 | 0 | 0 | 0 |
| *Table S4* | 166 | 0 | 0 | 1 | 0 | 0 |
| *-* | 57 | 0 | 1 | 1 | 0 | 0 |
| *Table S4* | 40 | 1 | 0 | 1 | 0 | 0 |
| *Table S4* | 33 | 0 | 0 | 1 | 1 | 0 |
| *-* | 16 | 1 | 0 | 0 | 1 | 0 |
| *-* | 14 | 0 | 1 | 0 | 1 | 0 |
| *-* | 10 | 1 | 1 | 0 | 0 | 0 |
| *Table S4* | 9 | 1 | 0 | 1 | 1 | 0 |
| *-* | 5 | 0 | 1 | 1 | 1 | 0 |
| *Table S6* | 2 | 0 | 0 | 0 | 0 | 1 |
| *-* | 2 | 1 | 1 | 1 | 0 | 0 |
| *-* | 1 | 1 | 1 | 1 | 1 | 0 |
| Total | 1253 *(Table S3)* | 360 | 363 | 313 | 420 | 2 |

**Supplementary Material 2** Characteristics of clients excluded from the study sample

Here we compared the excluded clients with the clients included in the study sample on observable characteristics, by means of four tables. Table S2 indicates which client records are included in each table.

In Table S3, the demographic factors of all excluded clients (N client records = 1,253) are shown. We found that the average age, the ratio of males to females and marital status were comparable between the study sample and excluded client records.

Table S4 displays the characteristics (if available) of all clients without care hours in the 28 days after the NANDA-I assessment. The case-mix group ‘Preventive care for frail elderly without current care need’ (PREV) was more prevalent in this group of clients than in the study sample. We consider this reasonable as this case-mix group entails clients that are given advice or instructions aimed at preventing further home healthcare use. These clients will not always receive actual home healthcare services. In our study sample, the clients in case-mix group PREV are also clients with lowest care hours.

Table S5 shows the excluded clients that did have registered nursing diagnoses but no registered case-mix group. The excluded clients have more diagnoses and more care hours on average. The most common NANDA-I diagnoses were largely similar between these clients and the clients in the study sample.

In Table S6, the characteristics of the excluded clients with registered case-mix group but without registered nursing diagnoses are shown. The average care hours within each case-mix group are similar between the included clients and this group of excluded clients. Compared to the study sample, we observed relatively many clients in case-mix groups PREV (4.6% compared to 0.7% in the study sample) and PALL (19.0% compared to 2.2% in the study sample). Both these case-mix groups are classified as short-term care groups, which might have reduced the likelihood of an extensive NANDA-I assessment by the district nurse.

**Table S3** Demographic factors for all excluded client records

| *N client records = 1,253* | | |  |
| --- | --- | --- | --- |
| Parameter | Class | Excluded clients | Clients in study sample |
|  |  | mean ± sd or N (%) | |
| Age |  | 75.5 ± 13.9 | 75.8 ± 13.9 |
| Gender | Male | 457 (36.5) | 1,598 (37.0) |
|  | Female | 796 (63.5) | 2,725 (63.0) |
| Marital status | Unknown | 874 (69.8) | 2,844 (65.8) |
|  | Unmarried | 49 (3.9) | 200 (4.6) |
|  | Married | 140 (11.2) | 570 (13.2) |
|  | Divorced | 12 (1.0) | 24 (0.6) |
|  | Widow(er) | 176 (14.0) | 677 (15.7) |
|  | Registered partnership | 2 (0.2) | 8 (0.2) |

**Table S4** Specifications for client records without care hours in 28 days after NANDA-I assessment

| *N client records = 248* |  |  |  |
| --- | --- | --- | --- |
| Parameter | Class | Excluded clients | Clients in study sample |
|  |  | mean ± sd or N (%) | |
| Case-mix group | *Not available* | *N = 42* |  |
|  | PREV | 38 (18.4) | 30 (0.7) |
|  | ST-H | 49 (23.8) | 694 (16.1) |
|  | ST-F | 42 (20.4) | 754 (17.4) |
|  | LT-SOM | 64 (31.1) | 2,359 (54.6) |
|  | LT-PG | 6 (2.9) | 389 (9.0) |
|  | PALL | 7 (3.4) | 97 (2.2) |
|  |  |  |  |
| NANDA-I characteristics | *Not available* | *N = 52* |  |
|  | Diagnoses | 2 ± 2 | 3 ± 3 |
|  | Symptoms | 8 ± 9 | 12 ± 13 |
|  | Etiologic/risk factors | 3 ± 3 | 4 ± 4 |

**Table S5** Specifications for client records with care hours in 28 days after NANDA-I assessment and with registered NANDA-I diagnoses

| *N client records = 342* | | |  |
| --- | --- | --- | --- |
| Parameter | Class | Excluded clients | Clients in study sample |
|  |  | mean ± sd or N (%) | |
| NANDA-I characteristics | Diagnoses | 5 ± 4 | 3 ± 3 |
|  | Symptoms | 23 ± 19 | 12 ± 13 |
|  | Etiologic/risk factors | 7 ± 6 | 4 ± 4 |
|  |  |  |  |
| Top five NANDA-I diagnoses |  | Risk for falls | Risk for falls |
|  |  | Ineffective health management | Ineffective health management |
|  |  | Impaired physical mobility | Impaired physical mobility |
|  |  | Bathing self-care deficit | Bathing self-care deficit |
|  |  | Chronic confusion | Dressing self-care deficit |
|  |  |  |  |
| Weekly home care hours | Average | 5.1 ± 5.4 | 3.0 ± 5.4 |

**Table S6** Specifications for client records with care hours in 28 days after NANDA-I assessment and with registered case-mix group

| *N client records = 284* | | |  |
| --- | --- | --- | --- |
| Parameter | Class | Excluded clients | Clients in study sample |
|  |  | mean ± sd or N (%) | |
| Case-mix group | PREV | 13 (4.6) | 30 (0.7) |
|  | ST-H | 43 (15.1) | 694 (16.1) |
|  | ST-F | 44 (15.5) | 754 (17.4) |
|  | LT-SOM | 108 (38.0) | 2,359 (54.6) |
|  | LT-PG | 20 (7.0) | 389 (9.0) |
|  | PALL | 54 (19.0) | 97 (2.2) |
|  | CHILD | 2 (0.7) | 0 (0.0) |
|  |  |  |  |
| Weekly home care hours | Average | 9.3 ± 16.3 | 3.0 ± 5.4 |
|  | PREV | 1.8 ± 3.0 | 1.2 ± 1.3 |
|  | ST-H | 1.4 ± 1.2 | 1.8 ± 2.0 |
|  | ST-F | 1.6 ± 1.1 | 1.9 ± 2.3 |
|  | LT-SOM | 3.5 ± 6.5 | 3.0 ± 3.9 |
|  | LT-PG | 2.9 ± 2.9 | 3.1 ± 3.6 |
|  | PALL | 37.6 ± 17.6 | 20.7 ± 22.5 |
|  | CHILD | *only two observations* | - |

**Supplementary Material 3**

**Fig S1** Decision tree for selecting the applicable case-mix group


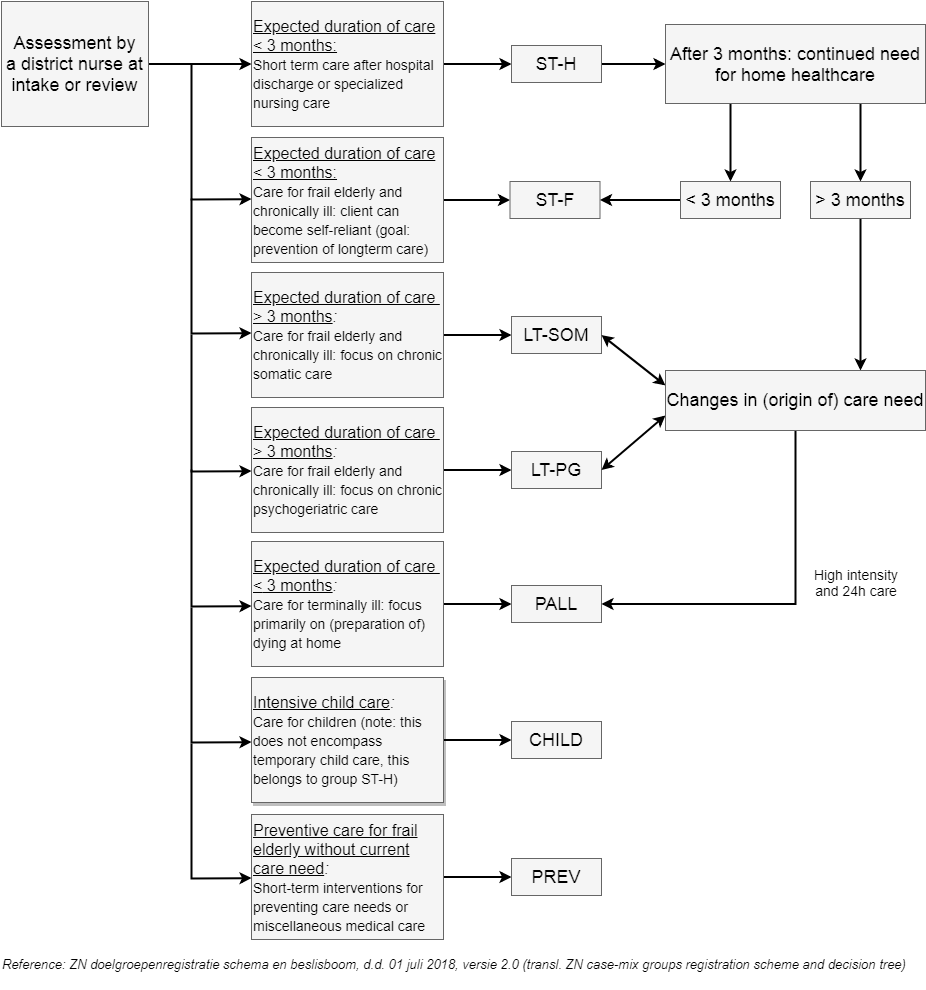


**Supplementary Material 4**

**Fig S2** Average home healthcare hours as a function of time, April 2017 - May 2018
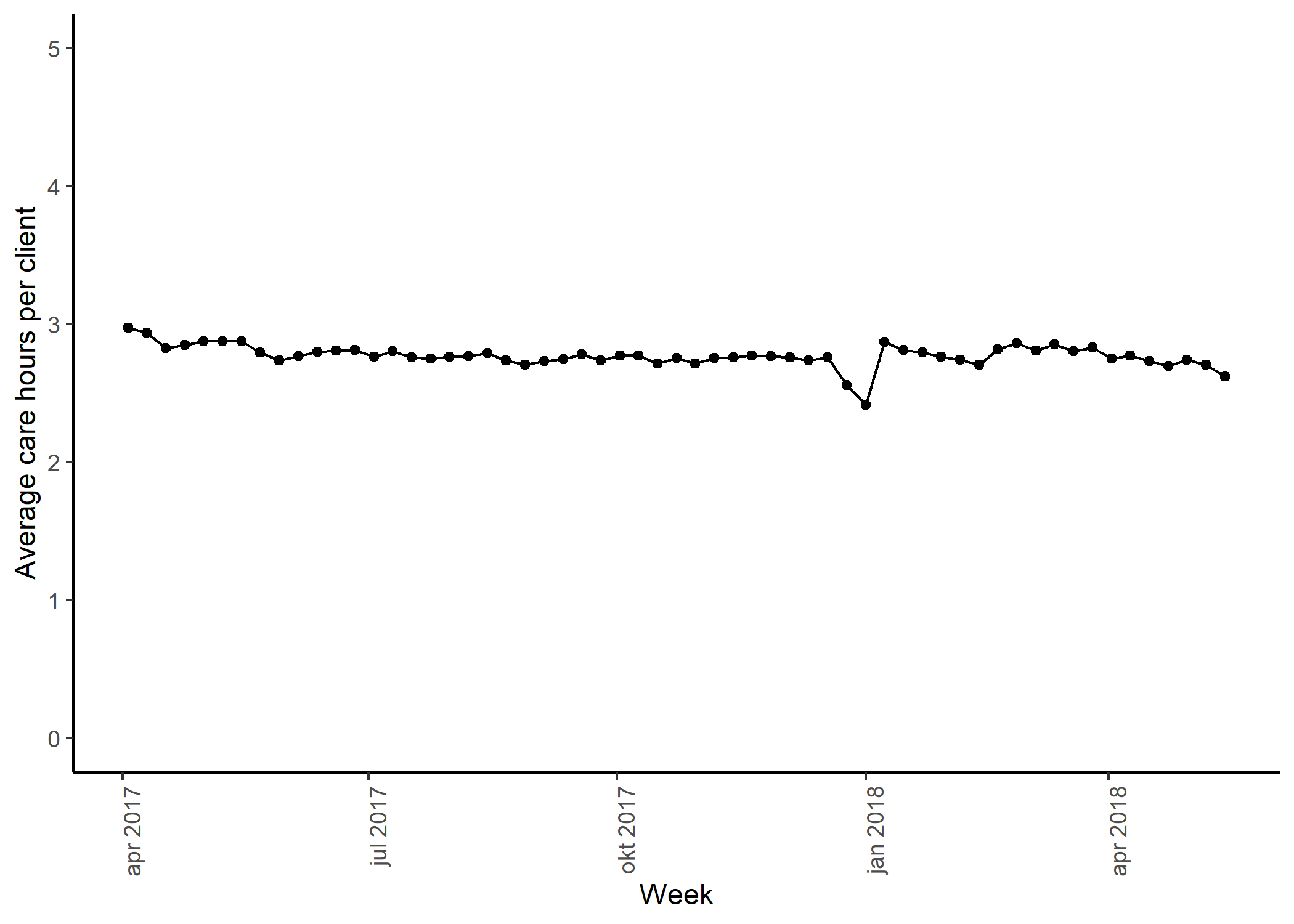


**Supplementary Material 5**

**Fig S3** Most common NANDA-I nursing diagnoses as a function of time, April 2017 - May 2018
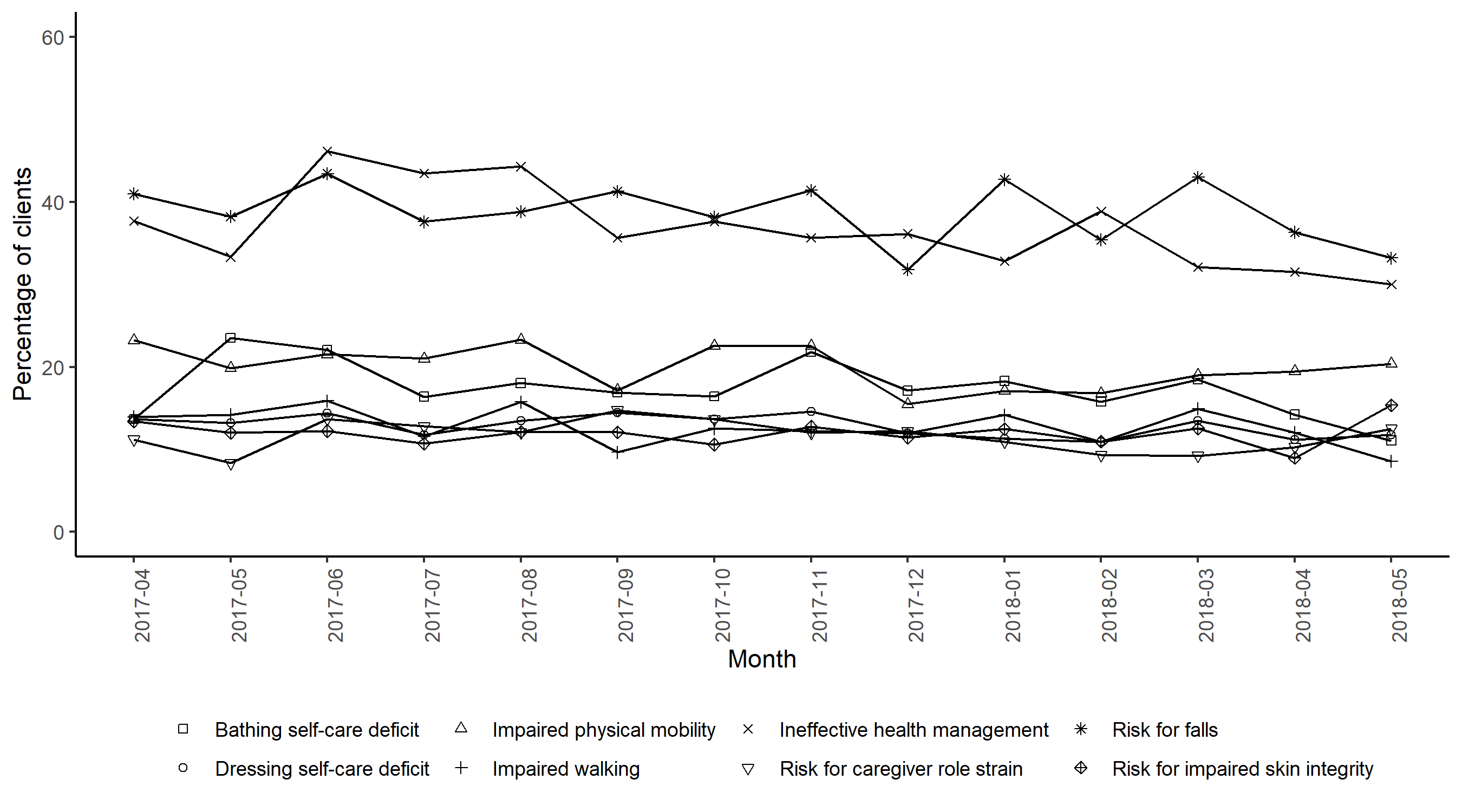


**Supplementary Material 6**

**Fig S4** Case-mix groups as a function of time, April 2017 - May 2018
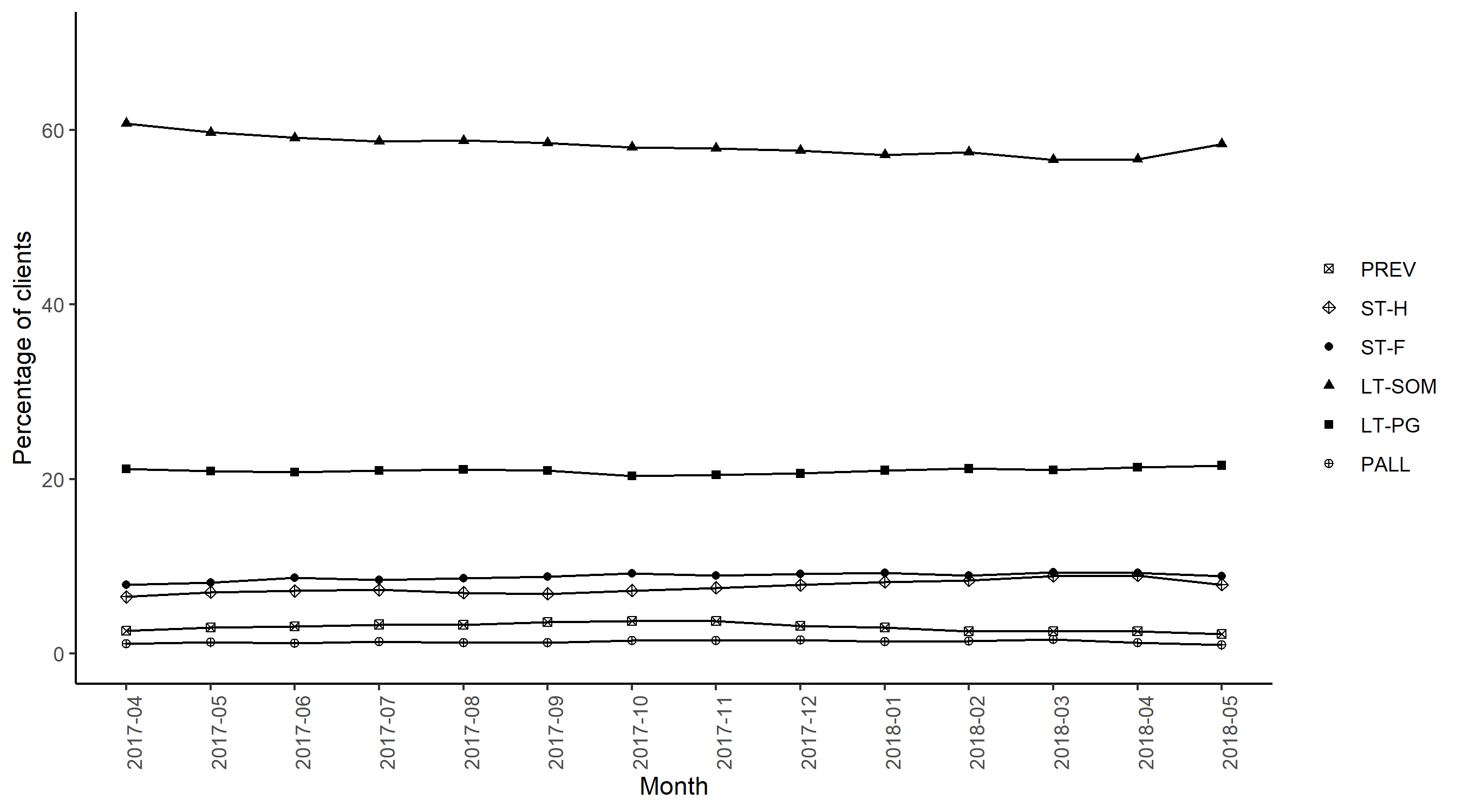


**Supplementary Material 7** Details on the methodology used

Random Forest is an ensemble machine learning algorithm that grows a large number of single de-correlated decision trees and averages them into a final prediction^1^. The algorithm does this by growing each *tree* over bootstrapped subsets of the data^1,2^. Also, at each split in each tree, only a limited number of predictors out of all predictors are randomly considered which also diversifies the trees^1,2^. Being a non-parametric algorithm, Random Forest is able to adapt to the data flexibly, without the need to specify functional form or interaction terms in advance^2^.

Random Forest is to a large extent immune to the presence of irrelevant variables in the model. However, if more irrelevant variables are added to a Random Forest model, at some point its performance will be adversely affected. We therefore performed recursive feature elimination (RFE) with Random Forest for all models in order to obtain models with optimal predictive performance. The rfe() function of the caret package in R was used, with the rangerFuncs set of helper functions^3^. Permutation importance was used as the variable importance mode. The Root Mean Squared Error (RMSE) was the performance metric used to choose the optimal subset size of the variables. Ten different subset sizes were tested by the RFE. These ranged from the first to the tenth decile of the number of variables when the model contained more than 10 variables; for models containing less than or exactly 10 variables, all possible subset sizes were considered.

The Random Forest implementation used (ranger package in R) includes several parameters that affect predictive performance. Tuning these parameters further optimizes the predictive power of the models. Three of the tuning parameters that apply to our algorithm were set at the default for regression: the minimum node size was 5, the splitting rule was the estimated response variance, and the number of trees was 500 for all models. For the latter, we verified whether the default value was sufficiently large by analyzing the OOB error rate as a function of the number of trees for the DEMO + CM + NANDA-I model and the total sample. As shown in the following plot, the OOB mean squared error rate had already settled down at lower values, which indicates that the number of trees was sufficient.


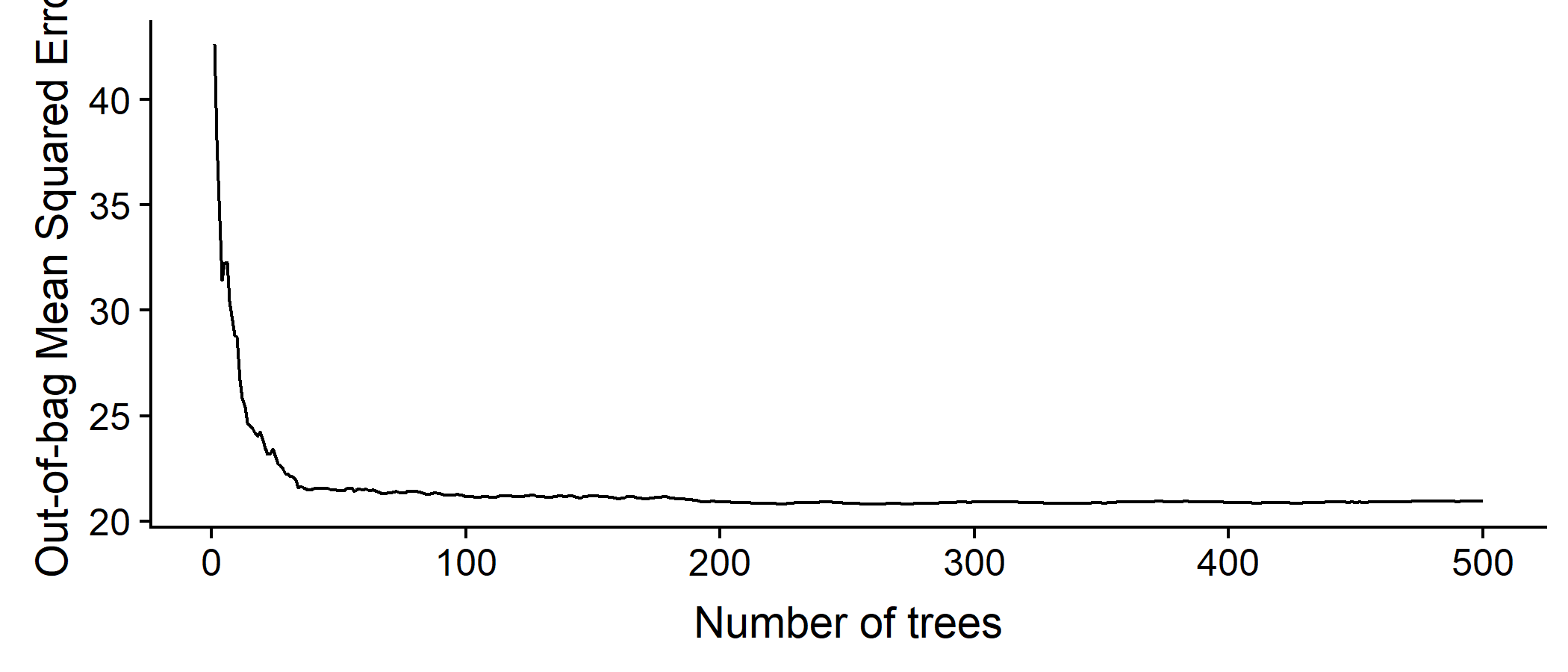


The number of variables available for splitting at each tree node (referred to here as *mtr*y) was separately tuned for all models. We varied mtry using a sequential doubling sequence ranging from only one variable up to the total number of selected variables in each model. The Random Forest algorithm chose the optimal mtry value for the final model. The selected mtry values for each total sample model were as follows:

| Model | N  variables | N variables selected through RFE | Sequence  for mtry selection | Selected  mtry |
| --- | --- | --- | --- | --- |
| DEMO | 3 | 1 (marital status) | 1 | 1 |
| DEMO + CM | 9 | 1 (PALL) | 1 | 1 |
| DEMO + NANDA-I | 391 | 157 | 1, 2, 4, 5, 8, 13, 19, 29, 44, 68, 103, 157 | 44 |
| DEMO + CM + NANDA-I | 397 | 41 | 1, 2, 3, 6, 12, 22, 41 | 12 |

References:

1. James G, Witten D, Hastie T, et al. *An introduction to statistical learning*. New York: Springer; 2013.
2. Chirikov VV, Shaya FT, Onukwugha E., et al. Tree-based Claims Algorithm for Measuring Pretreatment Quality of Care in Medicare Disabled Hepatitis C Patients. *Med Care.* 2017; 55(12):e104-e112.
3. Kuhn M, Johnson K. *Applied Predictive Modeling*. New York: Springer; 2013.

**Supplementary Material 8**

**Table S7** Summary of fit results for total study sample using OLS regression

| Model | N variables selected through RFE | MAPE  (hrs) | CPM  (%) | RMSE  (hrs) | R-squared  (%) |
| --- | --- | --- | --- | --- | --- |
| INT | 0 | 2,3 | 0 | 5,4 | 0 |
| DEMO | 3 | 2,3 | 0,4 | 5,4 | 0,3 |
| DEMO + CM | 7 | 2,1 | 8,7 | 4,7 | 23,8 |
| DEMO + NANDA-I | 40 | 2,3 | 0,8 | 5,2 | 2,9 |
| DEMO + CM + NANDA-I | 41 | 2,1 | 7,1 | 4,7 | 21,5 |

MAPE indicates Mean Absolute Prediction Error; CPM, Cumming’s Prediction Measure; RMSE, Root Mean Squared Error; hrs, hours

**Supplementary Material 9** Definitions of model performance measures

We use several measures of fit to determine the predictive performance of our models: the MAPE, CPM, RMSE and R-squared.

1. The MAPE is calculated as follows:
2. The CPM is calculated as follows:
3. The RMSE is calculated as follows:
4. The R-squared is calculated as follows:

**Supplementary Material 10**

**Table S8** Summary of fit results for clients in case-mix group PREV and case-mix group PALL

| Case-mix group | Model | N clients | MAPE (hrs) | CPM (%) | +/- sd^*^ (%) | RMSE (hrs) | R-squared (%) | +/- sd^*^ (%) |
| --- | --- | --- | --- | --- | --- | --- | --- | --- |
| PREV | INT | 30 | 0,8 | 0 | 0 | 1,3 | 0 | 0 |
|  | DEMO |  | 0,9 | -86,2 | 45,4 | 1,1 | -1008,8 | 1538,8 |
|  | DEMO + NANDA-I |  | 0,7 | -57,7 | 45,9 | 1,0 | -950,8 | 1546,5 |
| PALL | INT | 97 | 18,6 | 0 | 0 | 22,3 | 0 | 0 |
|  | DEMO |  | 18,3 | -0,6 | 1,8 | 22,3 | -5,3 | 2,2 |
|  | DEMO + NANDA-I |  | 14,9 | 18 | 1,4 | 18,6 | 24,5 | 2,7 |
| MAPE indicates Mean Absolute Prediction Error; CPM, Cumming’s Prediction Measure; RMSE, Root Mean Squared Error; hrs, hours; sd, standard deviation  *standard deviation is generated using 30 repeated 10-fold cross-validation | | | | | | | | |
